# K-mer-based approach for serodiagnostic antigen discovery in Chagas disease using unassembled sequencing reads

**DOI:** 10.1371/journal.pntd.0013835

**Published:** 2025-12-22

**Authors:** Nathan Ravi Medeiros Honorato, Laila Viana de Almeida, João Luís Reis-Cunha, Vanêssa Gomes Fraga, Daniel Menezes Souza, Ramon Vieira Nunes, Fátima Ribeiro-Dias, Edward Valencia Ayala, Jeffery A. Noble, Alexandre Ferreira Marques, M. G. Finn, Lúcia Maria da Cunha Galvão, Daniella C. Bartholomeu

**Affiliations:** 1 Departamento de Parasitologia, Instituto de Ciências Biológicas, Universidade Federal de Minas Gerais, Belo Horizonte, Minas Gerais, Brazil; 2 Department of Biology, University of York, York, Yorkshire, United Kingdom; 3 Departamento de Patologia Clínica, COLTEC, Universidade Federal de Minas Gerais, Belo Horizonte, Minas Gerais, Brazil; 4 Laboratório de Imunidade Natural (LIN), Instituto de Patologia Tropical e Saúde Pública, Universidade Federal de Goiás, Goiânia, Goiás, Brazil; 5 Laboratorio de Parasitología Molecular y Celular, Facultad de Ciencias Biológicas, Universidad Nacional Mayor de San Marcos, Lima, Peru; 6 School of Chemistry and Biochemistry, Georgia Institute of Technology, Atlanta, Georgia, United States of America; 7 Center for Molecular and Cellular Biosciences, School of Biological, Environmental, and Earth Sciences, University of Southern Mississippi, Hattiesburg, Mississippi, United States of America; 8 Departamento de Análises Clínicas e Toxicológicas, Universidade Federal do Rio Grande do Norte, Natal, Rio Grande do Norte, Brazil; The Ohio State University, UNITED STATES OF AMERICA

## Abstract

*Trypanosoma cruzi*, the causative agent of Chagas disease (CD), exhibits remarkable genetic diversity, classified into six discrete typing units (DTUs), and one additional DTU, TcBat, primarily associated with bats. These DTUs are distributed differentially across CD-endemic regions, posing significant challenges for molecular and serological diagnosis, as test performance often varies geographically. Identifying conserved genomic regions shared among parasites circulating in distinct endemic areas is therefore essential. However, complete or semi-complete genome assemblies are available for only a limited number of strains, insufficiently capturing intra- and inter-DTU variability, particularly within repetitive multigene families. A wealth of raw *T. cruzi* genomic reads is publicly available, offering an opportunity to investigate highly repetitive, high-copy number sequences that are difficult to assemble but potentially valuable for improving diagnostic sensitivity. In this study, we applied a read-based bioinformatics pipeline to analyze data from six DTUs (TcI–TcVI), generating 80-mer fragments and clustering them to identify conserved sequences. Consensus sequences from conserved clusters were used to design synthetic peptides, which were evaluated serologically with samples from chronically infected individuals from Brazil, Bolivia, and Peru. Four peptides from the conserved C-terminal region of mucin family proteins demonstrated robust diagnostic performance (AUC: 0.8783-0.9353), with particularly high values obtained with sera from Brazilian and Bolivian patients. Overall, our results demonstrate that k-mer-based, assembly-free approaches can successfully identify conserved antigens across genetically diverse *T. cruzi* populations, underscoring their value as discovery tools for potential serological markers. While the peptides identified here represent promising candidates, validation in larger and more geographically diverse cohorts will be essential to establish their broader diagnostic applicability. Importantly, similar genome-informed strategies may also be leveraged to guide the discovery of diagnostic targets for other infectious diseases.

## Author summary

Chagas disease is a neglected tropical disease caused by the parasite *Trypanosoma cruzi*, which shows a high level of genetic diversity across Latin America. This variation can affect the accuracy of diagnostic tests, especially in regions where the parasite has distinct genetic profiles. In this study, we analyzed publicly available DNA sequencing data from several isolates from multiple genetic groups of the parasite to identify regions that are conserved across different populations. Instead of assembling full genomes, we used a method based on clusterization of short DNA fragments, enabling the identification of conserved and high-copy number sequences across the parasite isolates—potential targets for improving diagnostic sensitivity. From these conserved regions, we designed small protein fragments (peptides) and tested them using blood samples from individuals with Chagas disease. Four peptides showed notable performance, particularly in samples from Brazil and Bolivia. Our findings support the use of bioinformatics tools to discover conserved targets in diverse parasite populations, contributing to the development of more reliable and regionally inclusive diagnostic tests for Chagas disease.

## 1. Introduction

Chagas disease, caused by the protozoan parasite *Trypanosoma cruzi*, is classified as a neglected tropical disease. It affects an estimated 6 to 7 million individuals, primarily in Latin America, with an additional 100 million people at risk of infection in endemic areas [1]. Furthermore, it accounts for over 10,000 deaths annually, posing a significant public health burden [2]. Recently, the United States was proposed as an endemic country for Chagas disease since several triatomine species can transmit the parasite to animals, including dogs, and invade human dwellings in the south [3]. The morbidity and mortality associated with *T. cruzi* extend beyond the Americas, impacting healthcare systems in non-endemic regions as well, owing to globalization and population mobility [1,4,5].

A critical obstacle to controlling the disease lies in the absence of fully reliable diagnostic tools that can effectively capture the diverse clinical and epidemiological scenarios of infection. The clinical manifestations of Chagas disease vary widely across individuals and disease stages, complicating accurate diagnosis. In particular, diagnostic challenges include the poor performance of current tests in detecting acute or congenital infections [6] and discrepancies in test results depending on the geographical origin of patients [7,8]. These issues are largely attributed to the extensive genomic and antigenic variability of *T. cruzi*, which hinders the development of universal diagnostic assays. In fact, *T. cruzi* is divided into at least seven discrete typing units (DTUs) from TcI to TcVI and Tcbat, exhibiting extensive inter- and intra-DTU diversity [9–11].

Due to the low and intermittent parasitemia during the chronic phase of *T. cruzi* infection, direct parasitological methods often lack sensitivity. As a result, serological approaches that detect anti-*T. cruzi* antibodies are considered more reliable for diagnostic purposes [6]. Over recent decades, several parasite surface proteins—such as trans-sialidases, Mucin-Associated Surface Proteins (MASPs), and mucins—have been identified as antigenic targets with potential for serological diagnosis [12–15]. However, the application of these antigens in immunodiagnosis remains challenging due to their extensive polymorphism among different *T. cruzi* strains and isolates [16–18]. Recent efforts employing high-density peptide arrays—screening approximately 2.8 million peptides derived from the CL Brener clone and the SylvioX10/1 strain—have shown promising diagnostic performance, with sensitivity and specificity above average [19]. Nevertheless, no single antigen or assay has yet achieved sufficient robustness to reliably confirm infection across diverse endemic regions using a standalone test.

Traditionally, diagnostic target discovery has relied heavily on reference strains maintained under prolonged in vitro culture [20], which may not accurately reflect the genetic and antigenic diversity of field isolates circulating in endemic areas. Additionally, the underrepresentation of antigenic repetitive regions in assembled genomes, along with the scarcity of fully assembled genomes despite the abundance of raw sequencing reads in public databases, hinders the identification of reliable targets for *T. cruzi* detection and underscores the importance of directly analyzing sequencing reads. In this context, a previous study from our group demonstrated that using peptides derived from short k-mers from *T. cruzi* sequencing reads enabled the detection of high-copy regions and conserved motifs derived from multigene families [21], and identified some potential trans-sialidase peptides that were recognized by sera from chronic chagasic patients. Building on this approach, the present study applied this strategy to identify conserved regions across *T. cruzi* field isolates from all DTUs—not only within polymorphic proteins but across the entire genome—that represent promising candidates for diagnostic targets capable of overcoming the challenges posed by *T. cruzi*’s extensive antigenic variability.

## 2. Methods

### Ethics statement

The use of human samples was approved by the Ethics Committee of the Federal University of Minas Gerais (protocol CAAE – 0559.0.203.000-11/2012). All subjects provided written informed consent before blood collection.

### DNA sequencing libraries selection and processing

Illumina whole-genome *T. cruzi* DNA libraries were retrieved from NCBI as follows: 25 from TcI, eight from TcII, one from TcIII, one from TcIV, one from TcV, and six from TcVI (**Table 1**). Selected libraries had at least 50% of their reads with an average Phred quality score of 25 and a minimum length of 50 nucleotides, as assessed by Trimmomatic [22]. Additionally, more than 70% of the trimmed reads had to be mapped to a *T. cruzi* reference genome using BWA-mem v0.7.12 [23]. The DNA libraries of DTUs TcI, TcIII, and TcIV were mapped against the SylvioX10/1 clone genome (from AnderssonLab; Genome Version/Assembly ID Mar 18, 2017); those of TcII against the YC6 clone genome (from GenBank; Genome Version/Assembly ID GCA_015033655.1); and those of TcV and TcVI against the CL Brener Esmeraldo-like haplotype clone (from GenBank; Genome Version/Assembly ID GCA_000209065.1). All references were obtained from version 62 of TriTrypDB (https://tritrypdb.org/tritrypdb/app/search/organism/GenomeDataTypes/result). Finally, only libraries that had a minimum genomic coverage of 20×, estimated using CADin (https://github.com/coqueiro-dos-santos/CADIn), were retained for subsequent analyses.

**Table 1:** Identification and metrics of *Trypanosoma cruzi* DNA read libraries.

Strain	SRA ID	DTU	Isolated from	Origin	Reads analyzed	Mapping (%)
Brazil_A4_2	SRR11803987	TcI	*Rattus rattus*	Brazil	28845913	74.04
TD23	SRR3676272	TcI	*Triatoma dimidiata*	Texas, USA	12306119	89.29
TD25	SRR3676273	TcI	*Triatoma dimidiata*	Texas, USA	16665089	87.28
X10462-P1C9	SRR3676274	TcI	*Homo sapiens*	Venezuela	14575070	86.38
X12422-P1C3	SRR3676275	TcI	*Homo sapiens*	Venezuela	14563715	84.92
CGl11	SRR3676276	TcI	*Homo sapiens*	Colombia	20102737	84.41
H6	SRR3676282	TcI	*Homo sapiens*	Panama	6914907	92.29
H7	SRR3676283	TcI	*Homo sapiens*	Panama	7708094	84.61
H12	SRR3676309	TcI	*Homo sapiens*	Panama	7789413	86.63
H14	SRR3676310	TcI	*Homo sapiens*	Panama	9262298	71.62
H15	SRR3676312	TcI	*Homo sapiens*	Panama	7779290	85.26
V1	SRR3676313	TcI	*Panstrongylus geniculatus*	Panama	8389411	83.56
V3	SRR3676315	TcI	*Triatoma dimidiata*	Panama	11984690	85.06
FcHcl2	SRR3676319	TcI	*Homo sapiens*	Colombia	12278323	85.19
TC127	SRR8503462	TcI	*Triatoma infestans*	Arequipa, Peru	12769552	70.24
TC134	SRR8503468	TcI	*Triatoma infestans*	Arequipa, Peru	13591494	71.54
TC023	SRR8503497	TcI	*Cavia porcellus*	Arequipa, Peru	16275405	70.90
TC041	SRR8503510	TcI	*Canis lupus familiaris*	Arequipa, Peru	16226626	74.96
TC044	SRR8503536	TcI	*Canis lupus familiaris*	Arequipa, Peru	14098102	71.38
TC050	SRR8503540	TcI	*Homo sapiens*	Arequipa, Peru	15987035	79.07
Bol-DH29	SRR8503542	TcI	*Homo sapiens*	Mairana, Bolivia	12893839	80.00
TC060	SRR8503559	TcI	*Panstrongylus lignarius*	Cajamarca, Peru	16100256	78.05
TC059	SRR8503560	TcI	*Panstrongylus lignarius*	Cajamarca, Peru	13374630	76.96
TC064	SRR8503574	TcI	*Cavia porcellus*	Arequipa, Peru	15656496	70.40
TC060-2	SRR8503577	TcI	*Panstrongylus lignarius*	Cajamarca, Peru	12438648	77.31
Ycl2_UFMG	SRR6357355	TcII	*Homo sapiens*	São Paulo, Brazil	25680066	85.74
S92a_UFMG	SRR6357356	TcII	*Homo sapiens*	Minas Gerais, Brazil	15597626	73.55
S44a_UFMG	SRR6357357	TcII	*Homo sapiens*	Minas Gerais, Brazil	15381991	80.47
S23b_UFMG	SRR6357358	TcII	*Homo sapiens*	Minas Gerais, Brazil	18045834	85.97
S162a_UFMG	SRR6357359	TcII	*Homo sapiens*	Minas Gerais, Brazil	20430071	82.87
S154a_UFMG	SRR6357360	TcII	*Homo sapiens*	Minas Gerais, Brazil	17585815	90.40
S15_UFMG	SRR6357361	TcII	*Homo sapiens*	Minas Gerais, Brazil	19593631	79.54
S11_UFMG	SRR6357362	TcII	*Homo sapiens*	Minas Gerais, Brazil	16490376	80.96
unespecified (231)	ERR864236	TcIII	*Homo sapiens*	Minas Gerais, Brazil	25661748	99.71
2886929923 (strain:CAN III cl.1)	SRR1996498	TcIV	*Homo sapiens*	Pará, Brazil	15173663	92.52
2886576934 (strain:92 80 cl.2)	SRR1996502	TcV	*Homo sapiens*	Santa Cruz, Bolivia	16863805	94.80
CLBrener_UFMG	SRR6357354	TcVI	*Triatoma infestans*	Rio Grande do Sul, Brazil	34949272	89.99
2875288070 (Tula cl2)	SRR831221	TcVI	*Homo sapiens*	Tulahuen, Chile	29542674	93.08
TC054	SRR8503551	TcVI	*Panstrongylus lignarius*	Cajamarca, Peru	17394647	72.82
TC052	SRR8503553	TcVI	*Panstrongylus lignarius*	Cajamarca, Peru	16307020	74.73
TC056	SRR8503557	TcVI	*Panstrongylus lignarius*	Cajamarca, Peru	15652188	75.78
TC062	SRR8503580	TcVI	*Cavia porcellus*	Cajamarca, Peru	16054446	73.75

### Nucleotide K-mer generation

For nucleotide k-mer generation, SAMtools v1.3.1 [24] was used to retrieve both mapped and unmapped DNA reads, avoiding the loss of repetitive sequences that might not be present in the assembled reference. The 80-nucleotide k-mers present in the selected reads were counted using Jellyfish v2.2.4 [25]. To avoid incorporating spurious k-mers generated by sequencing errors, only those with a minimum occurrence equal to 30% of the genomic coverage value of their source sample were included in the analyses. Additionally, to normalize nucleotide k-mer frequency between samples, these values were divided by the average genomic coverage of the corresponding libraries. In all runs, the hash table size was set to 275M (-s 275M), 10 threads were used, and the -C flag was used to report both the read sequence and its reverse complement in the output file (https://www.cbcb.umd.edu/software/jellyfish/jellyfish-manual-1.1.pdf). The dump command was then used to convert the nucleotide sequences to FASTA format.

### Nucleotide K-mer clustering and consensus generation

Subsequently, the nucleotide k-mers from all samples were combined into a single FASTA file and clustered using CD-HIT-EST v4.8.1 [26,27] with a 95% identity cut-off. During clustering, sequences were assigned to the first cluster that met the cut-off (-d 0), requiring the alignment to cover at least 97% of the sequence length (-aL 0.97 and -aS 0.97), using 16 CPU threads (-T 16) and 75 GB of RAM (-M 75000). Only clusters containing k-mers from all evaluated read libraries were considered conserved and included in the subsequent steps.

Next, MAFFT v7.427 [28], with the --adjustdirectionaccurately subcommand enabled, was used to perform multiple nucleotide sequence alignments, and then a consensus sequence for each cluster was generated using the consensus function from the seqinr v4.2.16 package [29] in R. A minimum relative frequency (threshold) of 90% was set to designate a nucleotide as the consensus at a given position. If this threshold was not met, an “N” was returned. Due to the alignment process, “N”s and “-” were frequently returned. When such an event occurred at the extremities of the sequences (5’ and 3’ ends), these characters were removed, reducing the sequence length. Consequently, sequences shorter than 70 nucleotides were excluded. Consensus sequences containing internal “N”s were also removed.

### Avoiding cross-reactivity

To reduce the probability of cross-reactivity in diagnostic tests, an initial Nucleotide BLAST (blastn) (https://blast.ncbi.nlm.nih.gov/Blast.cgi) was performed against the NCBI non-redundant nucleotide collection (nr/nt) using the following algorithm parameters: short queries selected, expected threshold word size of 0.05, max matches in a query range of 0, match/mismatch scores of 1,-2, gap costs linear, filter selecting low complexity regions, and mask selecting mask for lookup table only. The first 1,000 matches for each evaluated sequence were returned. Consensus nucleotide sequences with any exclusive match for *Trypanosoma cruzi* (taxid: 5693), *Trypanosoma cruzi marinkellei* (taxid: 85056), *Trypanosoma cruzi* subsp. *marinkellei* (taxid: 85056), *Trypanosoma cruzi dionisii* (taxid: 78083), or *Trypanosoma cruzi cruzi* (taxid: 85057) had their headers tagged with “tcruzi.” The remaining sequences were marked as “others.” Subsequently, to further reduce the probability of cross-reactivity, a 50% identity clustering was performed with the same parameters as described above. Clusters containing nucleotide sequences marked as “others” were excluded, while those composed only of sequences marked as “tcruzi” were retained.

Next, to identify the corresponding amino acid sequences and determine the proteins in which the consensus regions were located for subsequent experimental assays, the consensus sequences that passed this stage were subjected to a local blastx (v2.2.30) search against the proteomes of SylvioX10/1, Y C6, CL Brener Esmeraldo-like, and Non-Esmeraldo-like. Only matches with identity and coverage above 90% were considered. This tool was used with a relatively high cutoff value to ensure the correct identification of the reading frame of the nucleotide sequences to be translated. Then, a BED file was generated from the blastx output table, containing information on the sequence name, start and end coordinates of the match, and reference protein. The getfasta command from bedtools [30] was used to retrieve the amino acid sequences from the reference proteomes.

To serve as an additional filter to minimize potential cross-reactivity in the experimental assays, a local blastp was performed against the nr/nt database to further reduce cross-reactivity with other organisms. The parameters used were based on the online NCBI version: -task “blastp-short” -gapopen 9 -max_target_seqs 1000 -word_size 2 -matrix PAM30 -threshold 16 -comp_based_stats 0 -evalue 1000 -window_size 15 -num_threads 20. The processes of header tagging and clustering were also executed after this step. Finally, the selected peptides were fragmented into 15-amino-acid portions with a sliding window of 2 residues using the sliding command of SeqKit v0.12.0 [31]. Sequences shorter than 15 amino acids were not fragmented and were kept in their original form.

### Sera samples

We evaluated the antigenicity of peptides conserved among different DTUs using sera of 93 infected individuals and 97 healthy humans from Brazil (Minas Gerais), Bolivia (Cochabamba and Santa Cruz), and Peru (Cajamarca, La Joya, and Quequeña). No DTU identification assay was performed on the infected human samples. Furthermore, 21 humans with visceral leishmaniasis (VL) from Minas Gerais and Goiás, Brazil, were also included to assess cross-reactivity. The details of the sample origin locations are described in **Table 2**.

**Table 2:** Description of the country and region of origin of sera from chronically *Trypanosoma cruzi*-infected individuals, uninfected individuals, and those with visceral leishmaniasis used in the ELISA serological assays.

Country	Region	*T. cruzi* infected	Not infected	Visceral leishmaniasis
Brazil	Minas Gerais	66	57	21
Bolivia	Cochabamba	5	8	0
	Santa Cruz	8	8	0
Peru	Cajamarca	8	8	0
	La Joya	4	8	0
	Quequeña	2	8	0
**Total**		**93**	**97**	**21**

### Immunoblotting screening assay

A total of 1,020 peptides were selected and synthesized in two nitrocellulose membranes using the SPOT technique [32]. Eighteen peptides described by Ricci et al. (2023) [19] were added on each membrane as positive controls. The membranes were used for performing a screening immunoblotting with pools of sera from chronically infected, healthy, and VL-infected individuals from Brazil. Both synthesis and antigenicity assays were performed as previously described [21].

The densitometric value of each spot was calculated using ImageJ software [33] with the Protein Array Analyzer plug-in (http://image.bio.methods.free.fr/ImageJ/?Protein-Array-Analyzer-for-ImageJ.html). The minimum cut-off for reactivity was 17,672.72, based on the mean plus two standard deviations of the values from all spots on the membrane evaluated with samples from uninfected individuals. Peptides with values above the cut-off in assays with sera from *T. cruzi*-infected patients and below the cut-off with negative and VL-infected samples were considered promising markers in this screening and were selected for the next evaluation stage.

### Soluble peptides synthesis and characterization

The 12 selected peptides in the immunoblotting screening stage were synthesized as soluble peptides using the ResPep SL automatic synthesizer (Intavis) and characterized on a MALDI-TOF/TOF Autoflex IIITM (Bruker Daltonics), as described previously in Fantin et al. (2021) [34]. Mass spectrometry data for the four peptides with the best diagnostic parameters, demonstrating a high degree of purity, are shown in **S1 Fig**.

### Antigenicity ELISA assay

The performance of the 12 peptides was individually evaluated by ELISA. We also tested the samples with CL Brener epimastigote crude extract as a control of the reactivity of the assay. Each well of a Greiner Bio-One Half Area ELISA Microplate was coated with 100 µL of sodium carbonate buffer (pH 9.6) with 2 µg/mL of antigen, corresponding to 0.2 µg of free (unconjugated) peptide per well. The plates were incubated for 16 h at 37ºC, washed manually four times with PBS + 0.05% Tween 20 and then blocked with 100 µL of PBS + 5% BSA for 1 h at 37ºC. Then, samples were diluted 1:100 in PBS + 2.5% BSA, and the final volume of 25 µL was added in duplicate and incubated for 1 h at 37ºC. Afterwards, plates were washed as previously described and incubated with 25 µL of horseradish peroxidase-conjugated anti-human IgG (Sigma-Aldrich), diluted 1:10,000 in PBS + BSA 2.5%, for 1 h at 37ºC. Next, the plates were washed and incubated with 25 µL of 40 mM O-Phenylenediamine Dihydrochloride (Thermo Scientific) and 4 mM hydrogen peroxide (Sigma-Aldrich) diluted in 0.1M citric acid and 0.2 phosphate buffer for 15 minutes at room temperature in a dark room. Then, 25 µL of H_2_SO_4_ 2M were used to interrupt the reaction, and the absorbance was measured at 492 nm also in a Multiskan GO Microplate Spectrophotometer (Thermo Scientific). All samples were assayed in duplicate and showed a coefficient of variation lower than 20%. The cutoff for each antigen was calculated based on the Receiver Operating Characteristic (ROC) curve, with a 95% confidence interval and considering the Youden Index (sensitivity % + specificity % - 100) [35] as the metric to estimate the area under the curve, using the cutpointr v1.1.2 R package [36]. The calculation of the cutoff values was performed considering all samples together, resulting in a single cutoff for each antigen. Accuracy were also calculated.

The four top-performing peptide sequences have been registered with the Brazilian National Institute of Industrial Property (INPI) under patent number BR1020250019370.

### Visualization of the 3D structures of the peptides

To evaluate the three-dimensional structure of the selected proteins and their epitopes, the Protein Data Bank (pdb) file of the target was first obtained from the AlphaFold Protein Structure Database (https://alphafold.ebi.ac.uk/) using its accession code. When this file was not available, the protein sequence was predicted using ColabFold v1.5.5: AlphaFold2 with MMseqs2 (https://colab.research.google.com), always selecting the first of the five predictions provided. Protein modeling was performed in PyMOL v3.0.3 [37], where the pdb files were imported and the regions corresponding to the peptides evaluated by ELISA were identified.

## 3. Results

**Fig 1:**
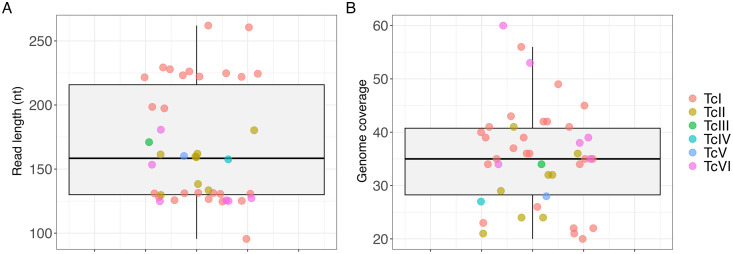
Comparison of read length (A) and genome coverage (B) across the evaluated *T. cruzi* read libraries. The dots over the boxplots represent the individual genomic libraries analyzed. Each color corresponds to a different DTU.

A total of 42 genomic read libraries from *T. cruzi* DTU I to VI, derived from a wide range of hosts, including vertebrates such as *Homo sapiens*, *Rattus rattus*, *Cavia porcellus*, and *Canis lupus familiaris*, as well as vectors such as *Triatoma infestans*, *Triatoma dimidiata*, *Panstrongylus lignarius*, and *Panstrongylus geniculatus*, were evaluated. These samples originated from geographically diverse regions, with representatives from North, Central, and South America, encompassing eight different countries. The number of reads that passed the trimming process and were used in subsequent steps varied greatly, ranging from 6,914,907 for TcI SRR3676282 to 80,954,115 for TcVI SRR6357354. Furthermore, the percentage of reads mapped to the assembled reference genomes (see Methods section) ranged from 70.24% to 99.71%, supporting the absence or low level of contamination by genetic material from organisms other than *T. cruzi* and/or distinct levels of sequence divergence or assembly completeness of the corresponding reference genome (**Table 1**). **Fig 1** presents some intrinsic characteristics of the libraries. The average read length ranged from 95.5 to 262 base pairs (**Fig 1A**), and sequencing coverage ranged from 21× to 60× (**Fig 1B**). It is important to note that, in all subsequent analyses, k-mer count values were normalized according to the respective genome coverage. Additional details can be found in **S1 Table**.

**Fig 2:**
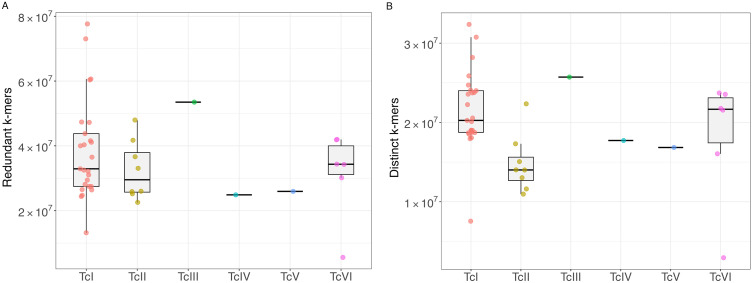
Evaluation of frequency and total number of k-mers derived from each analyzed sample. The number of redundant (A) and distinct (B) k-mers is shown. Count values were normalized by the respective genome coverage. The dots over the boxplots represent the genomic libraries analyzed. Each color corresponds to a different DTU.

For the analysis, k-mers were classified into two categories: redundant k-mers, which accounted for the overall count, and distinct k-mers, defined as unique sequences with no repetition. Regarding the total number of k-mers (redundant kmers) generated from the read libraries, it was observed that all DTUs yielded sequences on the order of 10^7^. TcI showed the most significant variation in the number of redundant sequences, whereas TcIII exhibited the highest median value. Since TcIII, TcIV, and TcV each had only one sample included in the analyses, their boxplots are represented as a single horizontal line (**Fig 2A**). When assessing the total number of distinct sequences used in this stage, the highest median value was found for DTU TcIII, with approximately 2.5 × 10^7^ k-mers, while the lowest was for TcII, with just under 1.5 × 10^7^ (**Fig 2B**). The group of TcI isolates showed the widest range of values for both redundant and distinct k-mers.

**Fig 3:**
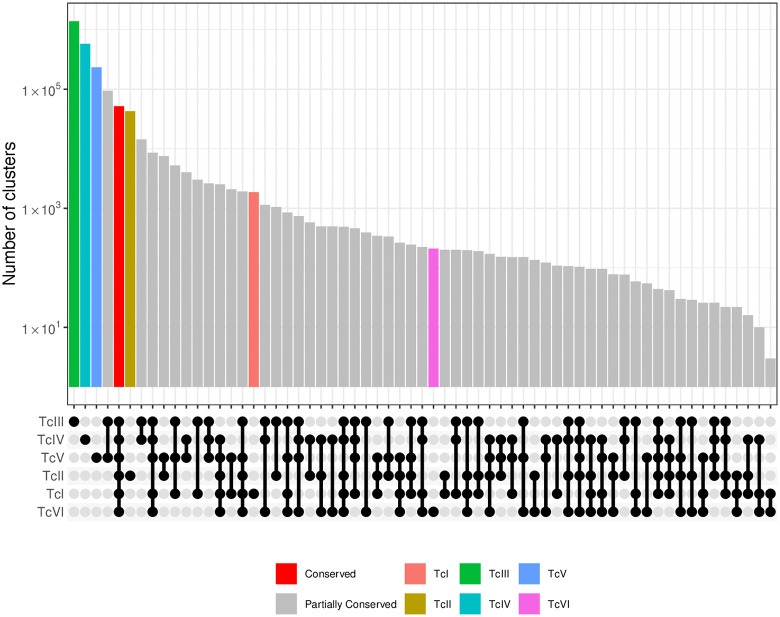
Upset plot showing the number of cluster sequences that are shared among different *T. cruzi* DTUs. The lower portion displays the genotypes involved in each shared set, indicated by dots and connecting lines. Bars represent the number of clusters for each sharing pattern. The y-axis is presented on a log_10_ scale.

The next analysis aimed to evaluate cluster sharing among the isolates. It is important to note that only clusters containing representatives from all samples of a given DTU were considered. For instance, to be classified as TcI-specific, a cluster had to include at least one sequence from each of the 25 read libraries assigned to the TcI genotype. In total, 2,455,821 clusters were identified, including DTU-specific clusters, clusters shared among DTUs, and clusters conserved across all samples. Among the latter, 51,772 clusters were found to be present in all 42 read libraries analyzed (**Fig 3**). After filtering out sequences that could potentially cause cross-reactivity in diagnostic assays, based on similarity to non-*T. cruzi* sequences, 543 consensus k-mer clusters were retained. These were conserved across all read libraries and exhibited a low risk of cross-reactivity. The resulting sequences were fragmented into 15-amino-acid peptides using a sliding window of two residues, synthesized onto nitrocellulose membranes, and subjected to a screening assay using sera from individuals in the chronic phase of Chagas disease as well as control sera.

**Fig 4:**
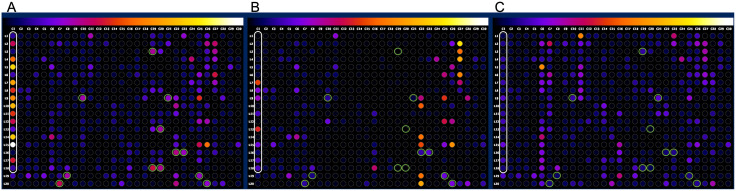
Antigenicity of peptides derived from conserved k-mer consensus sequences using different human samples. On the left (A) are individuals in the chronic phase of *T. cruzi* infection, in the center (B) are uninfected donors, and on the right (C) are individuals with visceral leishmaniasis. Each dot corresponds to a peptide synthesized on a nitrocellulose membrane. The reactivity of each peptide is represented on a scale ranging from black (low), orange (medium), to white (high). White ellipse represents the 18 peptides used as reactivity controls (see Methods section). Green circles indicate peptides whose densitometric values are above the cut-off (mean of negatives + 2* standard deviation of negatives) for infected individuals and below the cut-off for the other sample groups.

Of the 1,020 spots screened by immunoblotting, 12 (highlighted in green in **Fig 4**) met the selection criteria—namely, they were above the cut-off for samples from individuals with chronic Chagas disease and below the threshold for negative and VL samples. **Fig 4** displays only one of the two peptide membranes synthesized (membrane 1). The second membrane (membrane 2), which did not contain any peptides that met the cut-off criteria, is shown in **S2 Fig**. Densitometric values for selected, as well as for all other tested peptides against the pooled sera, are shown as boxplots in **S3 Fig**. It is worth noting that some non-selected spots in the Chagas group were also above the cut-off; however, these were excluded because they also showed high reactivity in negative and/or VL samples. In the end, 12 peptides were selected for soluble synthesis.

**Fig 5:**
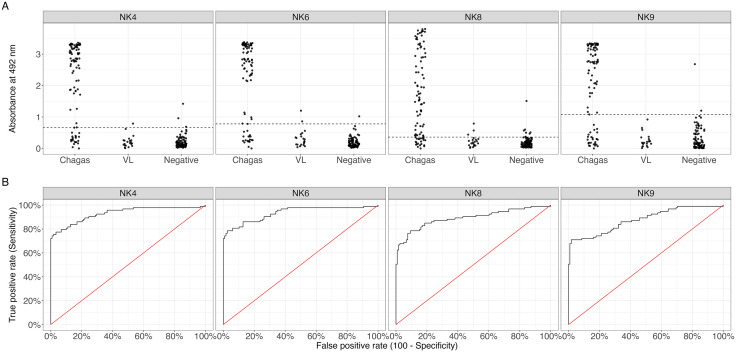
Recognition by human sera (A) and ROC curves (B) for the described peptides in ELISA assays using sera from individuals in the chronic phase of *T. cruzi* infection, from patients with VL, and uninfected donors. In **(A)**, each plot corresponds to one tested peptide. On the left of each plot are individuals infected with *T. cruzi* (Chagas), in the center, those with VL, and on the right, uninfected controls (Negative). The dashed line indicates the cut-off value based on the ROC curve and Youden’s index. VL: individuals with visceral leishmaniasis. In **(B)**, the black line represents the ROC curve generated from the serodiagnostic assays. The red diagonal line indicates the reference line where classification of infection status is random.

The next step was to evaluate the reactivity of the soluble peptides using human sera. Here, we present data only for the four best-performing peptides—NK4, NK6, NK8, and NK9. In the indirect ELISA, depending on the peptide, 66 to 73 of the 93 patients with chronic Chagas disease showed absorbance values above the cut-off (**Fig 5A**, **Table 3**). Conversely, 107 to 116 of the 118 non-infected (negative and VL groups) displayed values below the cut-off. The corresponding ROC curves, considering all populations combined, are shown in **Fig 5B**.

**Table 3:** Diagnostic parameters of ELISA for the four best soluble peptides derived from conserved k-mers.

Antigen	Region	*Cutoff*	TP	FP	TN	FN	SEN	SPE	Accuracy	AUC	Youden’s index
NK4	Bolivia	0.26	13	0	16	0	100.00	100.00	100.00	1	1
	Brazil	0.34	58	8	70	8	87.88	89.74	88.89	0.938	0.7762
	Peru	0.66	8	2	22	6	57.14	91.67	78.95	0.756	0.4881
	**Total**	**0.66**	**72**	**4**	**114**	**21**	**77.42**	**96.61**	**88.15**	**0.9317**	**0.7403**
NK6	Bolivia	0.26	13	0	16	0	100.00	100.00	100.00	1	1
	Brazil	0.79	53	2	76	13	80.30	97.44	89.58	0.9345	0.7774
	Peru	0.78	8	1	23	6	57.14	95.83	81.58	0.8304	0.5298
	**Total**	**0.78**	**73**	**3**	**115**	**20**	**78.49**	**97.46**	**89.10**	**0.9353**	**0.7595**
NK8	Bolivia	0.32	11	0	16	2	84.62	100.00	93.10	0.8606	0.8462
	Brazil	0.36	57	4	74	9	86.36	94.87	90.97	0.9476	0.8124
	Peru	0.71	5	1	23	9	35.71	95.83	73.68	0.5476	0.3155
	**Total**	**0.36**	**73**	**11**	**107**	**20**	**78.49**	**90.68**	**85.31**	**0.8937**	**0.6917**
NK9	Bolivia	0.75	10	0	16	3	76.92	100.00	89.66	0.8558	0.7692
	Brazil	1.16	51	0	78	15	77.27	100.00	89.58	0.9259	0.7727
	Peru	1.08	6	2	22	8	42.86	91.67	73.68	0.5119	0.3452
	**Total**	**1.08**	**66**	**2**	**116**	**27**	**70.97**	**98.31**	**86.26**	**0.8783**	**0.6927**

TP: true positives; FP: false positives; TN: true negatives; FN: false negatives; SEN: sensitivity: SPE: specificity; AUC: area under the curve.

**Fig 6:**
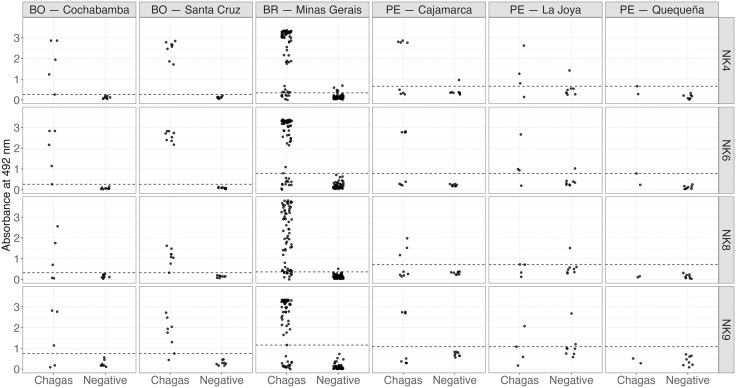
Recognition of soluble peptides by sera from individuals with chronic Chagas disease and uninfected individuals, according to geographic origin. On the left side of each plot are individuals with CD (Chagas), and on the right side are uninfected individuals (Negative). The dashed line indicates the cut-off value based on the ROC curve and Youden’s index.

When analyzed by geographic region, the majority of infected individuals were successfully detected, while almost all negative samples remained below the cut-off. The only exception was in Quequeña, Peru, where infected individuals showed absorbance values below the cut-off for both NK8 and NK9; for the NK4 and NK6 peptides, one sample was at the threshold (**Fig 6**). The performance of the peptides can be compared to that of the CL Brener epimastigote crude extract, as both exhibited similar overall absorbance values and reactivity patterns, including the low reactivity of Peruvian samples, as seen in the **S4 Fig**.

Considering the populations from all countries (**Table 2**), the parameters presented in **Table 3** show that peptides NK6 and NK8 exhibited the highest sensitivities (78.49%), while NK9 demonstrated the highest specificity (98.31%). The area under the curve (AUC) was also high for all peptides, with NK6 exhibiting the highest value of 0.9353 and NK9 the lowest at 0.8783. When stratifying the parameters by country of origin, Bolivia presented specificity of 100%, and Brazil and Peru displayed notably high specificity, with values above 89.74% for all peptides evaluated. However, the antigens were markedly less effective for individuals from Peru, with NK6 performing best in this group, showing a sensitivity of 57.14% and a specificity of 95.83% (**Table 3**).

**Fig 7:**
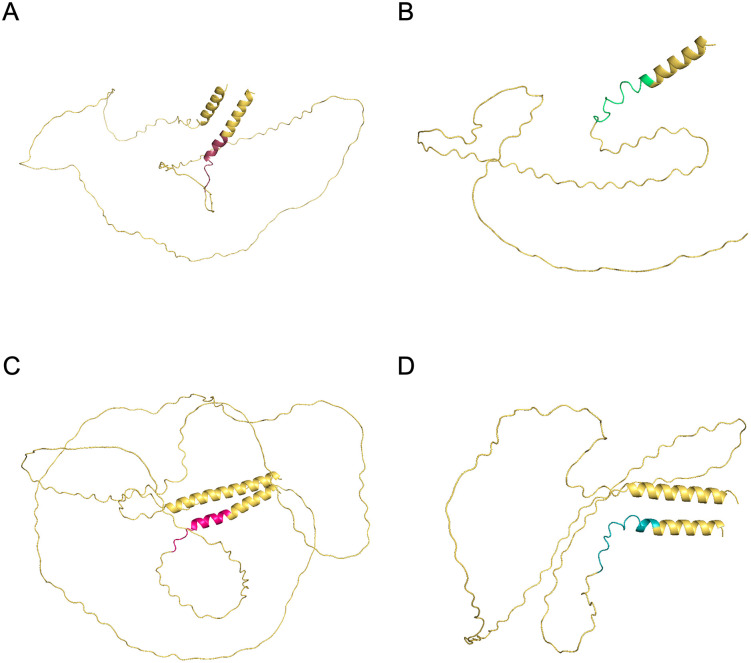
Mapping of peptides NK4 (A), NK6 (B), NK8 (C), and NK9 (D) on mucin proteins. The colored segments represent the peptides in each panel, while the yellow segments indicate the remaining regions of the corresponding proteins.

The blastx analysis of the original consensus sequences revealed that the four NK peptides with the best ELISA performance are derived from mucin superfamily. Peptides NK4, NK8, and NK9 matched proteins from the Y C6 strain and the CL Brener Esmeraldo-like clone, annotated as “mucin TcMUCII, putative.” Peptide NK6, in contrast, matched only a protein from the SylvioX10/1 strain, annotated as “Mucin-like glycoprotein” (**Table 4**). Notably, the antigenic regions are located at the C-terminal portions of the proteins. These regions correspond to a small segment of an alpha helix, preceded by a larger linear region (**Fig 7**).

**Table 4:** Identified peptides, sequences, genome references, and protein annotations across *Trypanosoma cruzi* strains.

Antigen	Peptide sequence	Genome reference	Protein code	Protein anotation	Protein size	Start	End
NK4	LREIDDSLSSSAWVC	Y C6	TcYC6_0151100	mucin TcMUC II, putative	237	207	221
			TcYC6_0151690	mucin TcMUC II, putative	192	162	176
		CL Brener Esmeraldo-like	TcCLB.507957.50	mucin TcMUC II, putative	237	207	221
NK6	LREIDGSLSSPAWVC	SylvioX10/1	TcSYL_0017600	Mucin-like glycoprotein	150	120	134
NK8	LREIDGSLSSSAWVF	Y C6	TcYC6_0150910	mucin TcMUC II, putative	221	191	205
		CL Brener Esmeraldo-like	TcCLB.510699.40	mucin TcMUC II, putative	373	343	357
			TcCLB.510371.130	mucin TcMUC II, putative	221	191	205
NK9	LRKIDGSFSSSAWVC	Y C6	TcYC6_0153700	mucin TcMUC II, putative	274	244	258
		CL Brener Esmeraldo-like	TcCLB.506667.70	mucin TcMUC II, putative	290	260	274
			TcCLB.508151.10	mucin TcMUC II, putative	217	187	201

## 4. Discussion

The serological diagnosis of *T. cruzi* infection presents several constraints that impact its accuracy [38]. The taxon exhibits extensive genetic variability, with marked inter- and intra-DTU antigenic variability [16–18], which reduces test sensitivity. Moreover, the distribution of DTUs across Latin America is heterogeneous [39], leading to regional differences in diagnostic performance [6,7,19]. Cross-reactivity further compromises specificity, as closely related parasites such as *Leishmania* and *T. rangeli*—frequently co-circulating in endemic regions—share conserved epitopes with *T. cruzi*. In addition, antigen discovery often relies on reference strains that fail to encompass the full spectrum of genetic variability present in field isolates. These challenges underscore the need for rational antigen design strategies aimed at optimizing both specificity and sensitivity.

In this study, we developed and applied a k-mer-based approach to analyze the complete genome sequencing read libraries of *T. cruzi* field isolates to identify potential serodiagnostic targets for Chagas disease. Using this strategy, we identified four promising peptides that are conserved across parasite DTUs and demonstrate high accuracy in distinguishing chagasic patients from healthy volunteers in Brazil, Bolivia, and Peru.

The current approach builds upon a read-based strategy originally developed by our group [21] to assess the variability and conservation of motifs derived from *T. cruzi* multigene families encoding surface proteins, extending its application to the whole genome. Other k-mer-based approaches have been employed in trypanosomatids for detecting hybridization events in *T. brucei* [40] and characterizing DTU-specific trans-sialidase sequences in *T. cruzi* [41]. The key strength of our methodology lies in exploiting the extensive availability of raw sequencing read libraries from field isolates in public repositories, in contrast to the relatively limited number of assembled genomes, most of which derive from reference strains. By not requiring gene-specific read mapping—often unreliable for multicopy genes—and not relying on genome assembly, the approach enables a comprehensive analysis of parasite genomic diversity directly from raw data. This is particularly advantageous given the highly repetitive and aneuploid nature of the *T. cruzi* genome, which hampers assembly accuracy and complicates variant identification [42,43]. Moreover, assembly accuracy differs across isolates, being influenced by genome repetitiveness, aneuploidy, sequencing technologies, and assembly strategies employed in each study; collectively, these factors hinder cross-genome comparisons and the consistent delineation of conserved regions suitable as diagnostic markers [43]. Leveraging the reduction in sequencing costs and increasing availability of field isolate data [44], we applied this k-mer-based framework to identify conserved markers across sequencing reads from isolates collected throughout Latin America.

Candidate regions were required to be conserved across all DTUs (TcI-TcVI), with particular emphasis on loci present in high copy numbers. We incorporated as many genomes as possible from each DTU; however, only a subset met the quality and completeness thresholds defined for our screening workflow. As a result, some DTUs—particularly DTUs III, IV, and V—were represented by only one assembly. This imbalance reflects a broader limitation of the current genomic landscape of *T.cruzi*, in which substantially more curated and complete genomes are available for TcI and TcII than for the remaining DTUs. Expanding the number of high-quality genomes, especially for DTUs III, IV, V, and VI, will be essential for more fully capturing the parasite’s genetic diversity and for strengthening the representativeness of genome-informed diagnostic approaches.

Further advances are also required to improve correlations between infecting DTUs and serological responses, including efforts to identify genotype-specific antigenic epitopes that could support the development of more discriminative serological tools. The absence of DTU identification for the human serum samples used in this study represents an additional limitation: the apparent broad reactivity of the peptides cannot be conclusively attributed to true antigenic conservation across all lineages and may instead reflect the DTU distribution within the clinical cohort. This challenge is inherent to Chagas disease research, as most patients are diagnosed during the chronic phase, when parasitemia is extremely low and parasite isolation or genotyping is often not feasible [45]. Continued methodological innovations—particularly approaches enabling reliable DTU assignment in chronic infections—will therefore be critical for establishing stronger links between parasite genetic background and serological performance.

To assess the serodiagnostic potential of the conserved sequences identified, we tested the corresponding peptides with sera from chronically infected individuals from Brazil, Bolivia, and Peru. The antigens demonstrated satisfactory performance in Brazilian and Bolivian samples, in some cases achieving >90% sensitivity and 100% specificity (**Table 3**). In contrast, all four evaluated peptides performed less effectively with sera from Peruvian individuals. The absence of these sequences in Peruvian strains may not fully account for this discrepancy, as La Joya and Quequeña—the districts from which the sera originated—are located in the Arequipa province, which was represented in the read libraries analyzed. Similar geographic discrepancies in immune responses have been reported previously. For instance, Martin et al. (2014) [46] documented lower IgG titers and fewer interferon-γ–secreting cells in Peruvian individuals compared to those from other South American regions, suggesting population-specific immunological variations. Additionally, another study analyzing antigen conservation across 14 assembled genomes reported that differences in antibody profiles among unequivocally positive and discordant serology cases were linked to individual patient variations, rather than to antigen variability, as the main factor impacting diagnostic performance [47]. Nevertheless, with regard to the Peruvian sera, our strategy achieved improved sensitivity, reaching 57.14%, compared to previously reported 30% using Stat Pack test [48]. Therefore, although population-specific immune responses may influence diagnostic performance, an antigen design strategy that accounts for the full spectrum of variability among parasite field isolates derived from distinct endemic regions is essential for developing universal diagnostic tests for Chagas disease.

Although this cohort represents the full set of samples available to this study, its size and geographic distribution were insufficient to support a formal statistical power calculation or to robustly assess regional differences in diagnostic performance. This constitutes an important limitation of this initial validation. Nevertheless, the inclusion of all accessible Brazilian samples and the complete set of international samples provides a meaningful preliminary overview of the expected performance of these antigens. Importantly, a larger and more geographically balanced cohort will be necessary to confirm the regional patterns observed here—particularly the lower reactivity detected in samples from Peru—and to draw more reliable conclusions regarding geographical variability.

Furthermore, the heterogeneous performance of the 18 control peptides included in the array – despite their prior validation in Ricci et al. (2023) [19] – highlights the marked antigenic complexity of *T. cruzi* and the influence of geographic and epidemiological context on antigen recognition. This divergence between studies underscores the importance of validation efforts incorporating field samples from diverse regions, as well as the value of strategies such as the genome-informed approach used here, which can systematically identify antigen candidates with broader and more reliable reactivity profiles. Future multicenter studies involving larger and more diverse serum panels across the Americans will therefore be essential to strengthen the generalizability and diagnostic applicability of these findings.

In this study, all peptides with promising diagnostic potential were derived from mucin proteins. Peptides NK4, NK8, and NK9 corresponded to sequences annotated as “TcMUC II, putative mucin” in the Y C6 and CL Brener-Esmeraldo-like genomes, whereas NK6 originated from a “mucin-like glycoprotein” in the Sylvio X10/1 strain. Mucins constitute the most abundant glycoproteins on the *T. cruzi* surface and are expressed throughout the parasite’s life cycle. TcMUC II, in particular, is predominantly expressed in trypomastigotes and is characterized by a long hypervariable central region followed by threonine-rich tandem repeats and a short conserved C-terminal domain located adjacent to the glycosylphosphatidylinositol anchor [49]. Notably, all four peptides identified in our screening mapped to this conserved C-terminal region, which explains their high degree of sequence identity. This domain is substantially less variable than the central tandem-repeat portion of TcMUCII and is consistently reported as immunogenic. Because our k-mer–based approach retrieved multiple peptides from this conserved region, several candidates displayed similar or nearly identical sequence motifs. Mucin proteins have previously been shown to elicit IgG responses in both humans and mice [50], and other mucin family members, such as TSSA, have likewise demonstrated strong potential as diagnostic antigens and as lineage-specific markers [3,12,40,51–53].

The use of antigens derived from repetitive regions, as identified here through k-mer analysis, represents a valuable diagnostic strategy. The TcCA-2 membrane protein, for instance, contains 12-amino-acid tandem repeats with minor sequence variations and has been demonstrated to be relevant for diagnosis of chronic infection [54]. One of these repeats exhibited high sensitivity and specificity, stronger reactivity in symptomatic compared to asymptomatic individuals, and potential utility as a marker for treatment efficacy [54,55]. Importantly, specific amino acid substitutions at critical positions can drastically alter diagnostic performance, in some cases reducing sensitivity from >90% to <5% [56]. As shown in **Table 4**, the peptides identified in this study display a high degree of conservation, with 10 of the 15 positions being invariant. Nonetheless, we observed variations in diagnostic performance among serum samples, underscoring how even subtle residue changes can affect antigenicity and diagnostic accuracy.

The peptides developed here demonstrated high specificity, avoiding cross-reactivity with other trypanosomatids or with vertebrate or invertebrate hosts. This is particularly relevant given that the epidemiological overlap of Chagas disease and leishmaniases poses a major diagnostic challenge, with cross-reactivity frequently reported. Shared antigens and genomic similarities between *Leishmania* spp. and *T. cruzi* can hinder accurate differential diagnosis [6,57,58]. A comparable limitation is observed with *T. rangeli*, a non-pathogenic species for humans that can nonetheless infect them, further undermining diagnostic accuracy [59,60]. Future studies should assess the performance of these markers against additional pathogens endemic to *T. cruzi* transmission areas, as well as in patients with non-infectious conditions that may interfere with conventional diagnostic methods. Nevertheless, the markers identified and validated here already exhibit strong potential for application. With further methodological refinement, they could substantially improve the accuracy and utility of these diagnostic tools in both research and public health contexts.

In summary, this study presents a novel strategy for the identification of serological markers of *T. cruzi* infection by exploiting conserved repetitive regions across diverse sequencing read libraries. The peptides derived from mucin proteins emerged as promising candidates, showing good specificity and encouraging diagnostic performance in our initial validation, particularly in the context of chronic infection. These findings highlight the value of bioinformatics-driven antigen discovery in addressing long-standing challenges in Chagas disease diagnosis, including cross-reactivity and parasite strain diversity, while also underscoring the need for broader validation. Importantly, understanding the infecting DTUs could substantially advance the field, enabling more refined investigations into potential serotyping strategies using field samples. Future studies incorporating larger, multicenter serum panels from additional geographic regions, as well as systematic assessments of cross-reactivity with other infections and conditions common in endemic populations, will be essential to fully establish the robustness and utility of these candidates. Beyond this contribution, our approach provides not only a framework for future innovations in serodiagnosis and disease surveillance across the Americas but also for the discovery of conserved antigenic targets in other infectious diseases where genomic diversity and cross-reactivity similarly hinder diagnostic development.

## Supporting information

S1 Fig Mass spectrometry analysis of the four peptides with the best diagnostic performance. The data were obtained by MALDI-TOF/TOF for NK4 (A), NK6 (B), NK8 (C) and NK9 (D), synthesized in soluble form.
(TIF)

S2 Fig Antigenicity of peptides derived from conserved k-mer consensus sequences using different human samples shown for membrane 2. No peptides on this membrane met the selection criteria (values above the cut-off for *T. cruzi*-infected individuals and below for negative and VL samples). Panel A shows individuals in the chronic phase of *T. cruzi* infection, panel B shows uninfected donors, and panel C shows individuals with visceral leishmaniasis. Each dot corresponds to a peptide synthesized on the nitrocellulose membrane. Reactivity is displayed on a color scale ranging from black (low), orange (medium), to white (high).
(TIF)

S3 Fig Boxplots of densitometric values for each peptide across the tested serum groups. The top panel shows the peptides selected for further analyses, which display a consistent pattern of values above the cut-off in Chagas sera and below the cut-off in visceral leishmaniasis (VL) and negative sera. The bottom panel shows all remaining peptides tested. Each boxplot represents one sample group: individuals infected with *T. cruzi* (left), those with VL (center), and uninfected individuals (right). The dashed line indicates the cut-off value (17,672.72). Points appearing above or below the boxes represent outliers, including occasional negative or VL samples with values exceeding the cut-off.
(TIF)

S4 Fig Recognition of soluble crude CL Brener epimastigotes (CE) by sera from individuals with chronic Chagas disease and uninfected individuals, according to geographic origin. On the left side of each plot are individuals with CD (Chagas), and on the right side are uninfected individuals (Negative). The dashed line indicates the cut-off value based on the ROC curve and Youden’s index.
(TIF)

S1 Table Other metrics of *Trypanosoma cruzi* genomic read libraries analyzed. (XLSX)

## References

[1] Echeverria LE, Morillo CA. American Trypanosomiasis (Chagas Disease). Infect Dis Clin North Am. 2019;33(1):119–34. doi: 10.1016/j.idc.2018.10.015 30712757

[2] World Health Organization (WHO). Time to integrate Chagas disease into primary health care. 2023; Available from: https://www.who.int/campaigns/world-chagas-disease-day/2023

[3] Beatty NL, Hamer GL, Moreno-Peniche B, Mayes B, Hamer SA. Chagas disease, an endemic disease in the United States. Emerg Infect Dis. 2025;31(9). 10.3201/eid3109.241700 PMC12407112 40866797

[4] Balouz V, Agüero F, Buscaglia CA. Chagas disease diagnostic applications: present knowledge and future steps. Adv Parasitol. 2017;97:1–45. doi: 10.1016/bs.apar.2016.10.001 28325368 PMC5363286

[5] Chatelain E. Chagas disease research and development: is there light at the end of the tunnel? Comput Struct Biotechnol J. 2016;15:98–103. doi: 10.1016/j.csbj.2016.12.002 28066534 PMC5196238

[6] Gomes YM, Lorena VMB, Luquetti AO. Diagnosis of Chagas disease: what has been achieved? What remains to be done with regard to diagnosis and follow up studies? Mem Inst Oswaldo Cruz. 2009;104 Suppl 1:115–21. doi: 10.1590/s0074-02762009000900017 19753466

[7] Caballero ZC, Sousa OE, Marques WP, Saez-Alquezar A, Umezawa ES. Evaluation of serological tests to identify Trypanosoma cruzi infection in humans and determine cross-reactivity with *Trypanosoma rangeli* and *Leishmania* spp. Clin Vaccine Immunol. 2007;14(8):1045–9. doi: 10.1128/CVI.00127-07 17522327 PMC2044488

[8] Guzmán-Gómez D, López-Monteon A, de la Soledad Lagunes-Castro M, Álvarez-Martínez C, Hernández-Lutzon MJ, Dumonteil E, et al. Highly discordant serology against Trypanosoma cruzi in central Veracruz, Mexico: role of the antigen used for diagnostic. Parasit Vectors. 2015;8:466. doi: 10.1186/s13071-015-1072-2 26384317 PMC4573690

[9] Lima L, Espinosa-Álvarez O, Ortiz PA, Trejo-Varón JA, Carranza JC, Pinto CM, et al. Genetic diversity of Trypanosoma cruzi in bats, and multilocus phylogenetic and phylogeographical analyses supporting Tcbat as an independent DTU (discrete typing unit). Acta Trop. 2015;151:166–77. doi: 10.1016/j.actatropica.2015.07.015 26200788

[10] Zingales B, Andrade SG, Briones MRS, Campbell DA, Chiari E, Fernandes O, et al. A new consensus for Trypanosoma cruzi intraspecific nomenclature: second revision meeting recommends TcI to TcVI. Mem Inst Oswaldo Cruz. 2009;104(7):1051–4. doi: 10.1590/s0074-02762009000700021 20027478

[11] Zingales B, Macedo AM. Fifteen years after the definition of Trypanosoma cruzi DTUs: what have we learned? Life (Basel). 2023;13(12):2339. doi: 10.3390/life13122339 38137940 PMC10744745

[12] Buchovsky AS, Campetella O, Russomando G, Franco L, Oddone R, Candia N, et al. trans-sialidase inhibition assay, a highly sensitive and specific diagnostic test for Chagas’ disease. Clin Diagn Lab Immunol. 2001;8(1):187–9. doi: 10.1128/CDLI.8.1.187-189.2001 11139217 PMC96032

[13] Cimino RO, Rumi MM, Ragone P, Lauthier J, D’Amato AA, Quiroga IRL, et al. Immuno-enzymatic evaluation of the recombinant TSSA-II protein of *Trypanosoma cruzi* in dogs and human sera: a tool for epidemiological studies. Parasitology. 2011;138(8):995–1002. doi: 10.1017/S0031182011000540 21518468

[14] dos Santos SL, Freitas LM, Lobo FP, Rodrigues-Luiz GF, Mendes TADO, Oliveira ACS. The MASP family of Trypanosoma cruzi: changes in gene expression and antigenic profile during the acute phase of experimental infection. PLoS Negl Trop Dis. 2012;6(8):e1779. doi: 10.1371/journal.pntd.0001779 PMC3419193 22905275

[15] Russomando G, Sánchez Z, Meza G, de Guillen Y. Shed acute-phase antigen protein in an ELISA system for unequivocal diagnosis of congenital Chagas disease. Expert Rev Mol Diagn. 2010;10(6):705–7. doi: 10.1586/erm.10.70 20843193

[16] Bartholomeu DC, Cerqueira GC, Leão ACA, daRocha WD, Pais FS, Macedo C, et al. Genomic organization and expression profile of the mucin-associated surface protein (masp) family of the human pathogen Trypanosoma cruzi. Nucleic Acids Res. 2009;37(10):3407–17. doi: 10.1093/nar/gkp172 19336417 PMC2691823

[17] El-Sayed NM, Myler PJ, Bartholomeu DC, Nilsson D, Aggarwal G, Tran AN. The genome sequence of *Trypanosoma cruzi*, etiologic agent of Chagas disease. Science. 2005;309(5733):409–15. 16020725 10.1126/science.1112631

[18] Seco-Hidalgo V, De Pablos LM, Osuna A. Transcriptional and phenotypical heterogeneity of *Trypanosoma cruzi* cell populations. Open Biol. 2015;5(12):150190. doi: 10.1098/rsob.150190 26674416 PMC4703061

[19] Ricci AD, Bracco L, Salas-Sarduy E, Ramsey JM, Nolan MS, Lynn K. The Trypanosoma cruzi antigen and epitope atlas: antibody specificities in Chagas disease patients across the Americas. Nat Commun. 2023;14(1):1850. 37012236 10.1038/s41467-023-37522-9 PMC10070320

[20] Moreira OC, Fernandes AG, Gomes NLDS, Dos Santos CM, Jacomasso T, Costa ADT, et al. Validation of the NAT Chagas IVD kit for the detection and quantification of Trypanosoma cruzi in blood samples of patients with Chagas disease. Life. 2023;13(6):1236. 37374019 10.3390/life13061236 PMC10300704

[21] Reis-Cunha JL, Coqueiro-Dos-Santos A, Pimenta-Carvalho SA, Marques LP, Rodrigues-Luiz GF, Baptista RP. Accessing the variability of multicopy genes in complex genomes using unassembled next-generation sequencing reads: the case of Trypanosoma cruzi multigene families. mBio. 2022;13(6):e0231922. doi: 10.1128/mbio.02319-22 PMC9765020 36264102

[22] Bolger AM, Lohse M, Usadel B. Trimmomatic: a flexible trimmer for Illumina sequence data. Bioinformatics. 2014;30(15):2114–20. 24695404 10.1093/bioinformatics/btu170 PMC4103590

[23] Li H. Aligning sequence reads, clone sequences and assembly contigs with BWA-MEM. 2013 [cited 2023 Jan 15]. Available from: https://arxiv.org/abs/1303.3997

[24] Li H, Handsaker B, Wysoker A, Fennell T, Ruan J, Homer N, et al. The sequence alignment/map format and SAMtools. Bioinformatics. 2009;25(16):2078–9. doi: 10.1093/bioinformatics/btp352 19505943 PMC2723002

[25] Marçais G, Kingsford C. A fast, lock-free approach for efficient parallel counting of occurrences of k-mers. Bioinformatics. 2011;27(6):764–70. doi: 10.1093/bioinformatics/btr034 21217122 PMC3051319

[26] Fu L, Niu B, Zhu Z, Wu S, Li W. CD-HIT: accelerated for clustering the next-generation sequencing data. Bioinformatics. 2012;28(23):3150–2. doi: 10.1093/bioinformatics/bts565 23060610 PMC3516142

[27] Li W, Godzik A. Cd-hit: a fast program for clustering and comparing large sets of protein or nucleotide sequences. Bioinformatics. 2006;22(13):1658–9. 16731699 10.1093/bioinformatics/btl158

[28] Katoh K, Standley DM. MAFFT multiple sequence alignment software version 7: improvements in performance and usability. Mol Biol Evol. 2013;30(4):772–80. doi: 10.1093/molbev/mst010 23329690 PMC3603318

[29] Charif D, Lobry JR. SeqinR 1.0-2: A Contributed Package to the R Project for Statistical Computing Devoted to Biological Sequences Retrieval and Analysis. In: Bastolla U, Porto M, Roman HE, Vendruscolo M, Greenbaum E, editors. Structural Approaches to Sequence Evolution (Biological and Medical Physics, Biomedical Engineering). Berlin, Heidelberg: Springer Berlin Heidelberg; 2007. pp. 207–32. [cited 2023 Jan 15] Available from: http://link.springer.com/10.1007/978-3-540-35306-5_10

[30] Quinlan AR, Hall IM. BEDTools: a flexible suite of utilities for comparing genomic features. Bioinformatics. 2010;26(6):841–2. 20110278 10.1093/bioinformatics/btq033 PMC2832824

[31] Shen W, Le S, Li Y, Hu F. SeqKit: a cross-platform and ultrafast toolkit for FASTA/Q file manipulation. PLoS One. 2016;11(10):e0163962. doi: 10.1371/journal.pone.0163962 27706213 PMC5051824

[32] Frank R. Spot-synthesis: an easy technique for the positionally addressable, parallel chemical synthesis on a membrane support. Tetrahedron. 1992;48(42):9217–32. doi: 10.1016/s0040-4020(01)85612-x

[33] Schneider CA, Rasband WS, Eliceiri KW. NIH Image to ImageJ: 25 years of image analysis. Nat Methods. 2012;9(7):671–5. doi: 10.1038/nmeth.2089 22930834 PMC5554542

[34] Fantin RF, Fraga VG, Lopes CA, De Azevedo IC, Reis-Cunha JL, Pereira DB. New highly antigenic linear B cell epitope peptides from PvAMA-1 as potential vaccine candidates. PLoS ONE. 2021;16(11):e0258637. doi: 10.1371/journal.pone.0258637 PMC8562794 34727117

[35] Lai C-Y, Tian L, Schisterman EF. Exact confidence interval estimation for the Youden index and its corresponding optimal cut-point. Comput Stat Data Anal. 2012;56(5):1103–14. doi: 10.1016/j.csda.2010.11.023 27099407 PMC4834986

[36] Thiele C, Hirschfeld G. Cutpointr: improved estimation and validation of optimal cutpoints in R. J Stat Soft. 2021;98(11).

[37] Schrödinger L, DeLano W. PyMOL. 2020. Available from: http://www.pymol.org/pymol

[38] Balouz V, Bracco L, Ricci AD, Romer G, Agüero F, Buscaglia CA. Serological approaches for Trypanosoma cruzi strain typing. Trends Parasitol. 2021;37(3):214–25. doi: 10.1016/j.pt.2020.12.002 33436314 PMC8900812

[39] Velásquez-Ortiz N, Herrera G, Hernández C, Muñoz M, Ramírez JD. Discrete typing units of Trypanosoma cruzi: geographical and biological distribution in the Americas. Sci Data. 2022;9(1):360. doi: 10.1038/s41597-022-01452-w 35750679 PMC9232490

[40] Kay C, Peacock L, Williams TA, Gibson W. Signatures of hybridization in Trypanosoma brucei. PLoS Pathog. 2022;18(2):e1010300. doi: 10.1371/journal.ppat.1010300 35139131 PMC8863249

[41] Probst CM, Melo MDFAD, Pavoni DP, Toledo MJDO, Galdino TS, Brandão AA, et al. A new Trypanosoma cruzi genotyping method enables high resolution evolutionary analyses. Mem Inst Oswaldo Cruz. 2021;116:e200538. doi: 10.1590/0074-02760200538 34468503 PMC8405150

[42] Arner E, Kindlund E, Nilsson D, Farzana F, Ferella M, Tammi MT, et al. Database of Trypanosoma cruzi repeated genes: 20,000 additional gene variants. BMC Genomics. 2007;8:391. doi: 10.1186/1471-2164-8-391 17963481 PMC2204015

[43] Majeau A, Murphy L, Herrera C, Dumonteil E. Assessing Trypanosoma cruzi parasite diversity through comparative genomics: implications for disease epidemiology and diagnostics. Pathogens. 2021;10(2):212. doi: 10.3390/pathogens10020212 33669197 PMC7919814

[44] Wetterstrand KA. National Human Genome Research Institute. DNA Sequencing Costs: Data from the NHGRI Genome Sequencing Program (GSP). 2023 [cited 2025 Jul 31]. Available from: www.genome.gov/sequencingcostsdata

[45] Schijman AG, Alonso-Padilla J, Britto C, Herrera Bernal CP. Retrospect, advances and challenges in Chagas disease diagnosis: a comprehensive review. Lancet Reg Health Am. 2024;36:100821. doi: 10.1016/j.lana.2024.100821 39006126 PMC11246061

[46] Martin DL, Marks M, Galdos-Cardenas G, Gilman RH, Goodhew B, Ferrufino L, et al. Regional variation in the correlation of antibody and T-cell responses to Trypanosoma cruzi. Am J Trop Med Hyg. 2014;90(6):1074–81. doi: 10.4269/ajtmh.13-0391 24710614 PMC4047731

[47] Majeau A, Dumonteil E, Herrera C. Identification of highly conserved Trypanosoma cruzi antigens for the development of a universal serological diagnostic assay. Emerg Microbes Infect. 2024;13(1):2315964. doi: 10.1080/22221751.2024.2315964 38381980 PMC10883094

[48] Verani JR, Seitz A, Gilman RH, LaFuente C, Galdos-Cardenas G, Kawai V, et al. Geographic variation in the sensitivity of recombinant antigen-based rapid tests for chronic Trypanosoma cruzi infection. Am J Trop Med Hyg. 2009;80(3):410–5. doi: 10.4269/ajtmh.2009.80.410 19270291

[49] Buscaglia CA, Campo VA, Di Noia JM, Torrecilhas ACT, De Marchi CR, Ferguson MAJ, et al. The surface coat of the mammal-dwelling infective trypomastigote stage of Trypanosoma cruzi is formed by highly diverse immunogenic mucins. J Biol Chem. 2004;279(16):15860–9. doi: 10.1074/jbc.M314051200 14749325

[50] De Pablos LM, Díaz Lozano IM, Jercic MI, Quinzada M, Giménez MJ, Calabuig E, et al. The C-terminal region of Trypanosoma cruzi MASPs is antigenic and secreted via exovesicles. Sci Rep. 2016;6:27293. doi: 10.1038/srep27293 27270330 PMC4897614

[51] Bhattacharyya T, Brooks J, Yeo M, Carrasco HJ, Lewis MD, Llewellyn MS, et al. Analysis of molecular diversity of the Trypanosoma cruzi trypomastigote small surface antigen reveals novel epitopes, evidence of positive selection and potential implications for lineage-specific serology. Int J Parasitol. 2010;40(8):921–8. doi: 10.1016/j.ijpara.2010.01.002 20097201

[52] De Marchi CR, Di Noia JM, Frasch ACC, Amato Neto V, Almeida IC, Buscaglia CA. Evaluation of a recombinant Trypanosoma cruzi mucin-like antigen for serodiagnosis of Chagas’ disease. Clin Vaccine Immunol. 2011;18(11):1850–5. doi: 10.1128/CVI.05289-11 21880857 PMC3209031

[53] Izquierdo L, Marques AF, Gállego M, Sanz S, Tebar S, Riera C, et al. Evaluation of a chemiluminescent enzyme-linked immunosorbent assay for the diagnosis of Trypanosoma cruzi infection in a nonendemic setting. Mem Inst Oswaldo Cruz. 2013;108(7):928–31. doi: 10.1590/0074-0276130112 24271047 PMC3970649

[54] Buschiazzo A, Campetella OE, Macina RA, Salceda S, Frasch AC, Sanchez DO. Sequence of the gene for a Trypanosoma cruzi protein antigenic during the chronic phase of human Chagas disease. Mol Biochem Parasitol. 1992;54(1):125–8. doi: 10.1016/0166-6851(92)90105-s 1518528

[55] Fernandez-Gomez R, Esteban S, Gomez-Corvera R, Zoulika K, Ouaissi A. Trypanosoma cruzi: Tc52 released protein-induced increased expression of nitric oxide synthase and nitric oxide production by macrophages. J Immunol. 1998;160(7):3471–9. doi: 10.4049/jimmunol.160.7.3471 9531308

[56] Thomas MC, Fernández-Villegas A, Carrilero B, Marañón C, Saura D, Noya O, et al. Characterization of an immunodominant antigenic epitope from Trypanosoma cruzi as a biomarker of chronic Chagas’ disease pathology. Clin Vaccine Immunol. 2012;19(2):167–73. doi: 10.1128/CVI.05566-11 22155766 PMC3272920

[57] Brandão EMV, Xavier SCC, Rocha FL, Lima CFM, Candeias ÍZ, Lemos FG, et al. Wild and domestic canids and their interactions in the transmission cycles of Trypanosoma cruzi and Leishmania spp. in an area of the Brazilian Cerrado. Pathogens. 2020;9(10):818. doi: 10.3390/pathogens9100818 33036238 PMC7600672

[58] Vexenat ADC, Santana JM, Teixeira AR. Cross-reactivity of antibodies in human infections by the kinetoplastid protozoa Trypanosoma cruzi, Leishmania chagasi and Leishmania (viannia) braziliensis. Rev Inst Med Trop Sao Paulo. 1996;38(3):177–85. doi: 10.1590/s0036-46651996000300003 9163981

[59] Dario MA, Pavan MG, Rodrigues MS, Lisboa CV, Kluyber D, Desbiez ALJ, et al. Trypanosoma rangeli genetic, mammalian hosts, and geographical diversity from five brazilian biomes. Pathogens. 2021;10(6):736. doi: 10.3390/pathogens10060736 34207936 PMC8230690

[60] de Moraes MH, Guarneri AA, Girardi FP, Rodrigues JB, Eger I, Tyler KM, et al. Different serological cross-reactivity of Trypanosoma rangeli forms in Trypanosoma cruzi-infected patients sera. Parasit Vectors. 2008;1(1):20. doi: 10.1186/1756-3305-1-20 18611261 PMC2475519

