# Supplementary figures and images for: K-mer-based approach for serodiagnostic antigen discovery in Chagas disease using unassembled sequencing reads
Source: PLoS Negl Trop Dis. 2025 Dec 22;19(12):e0013835. doi: 10.1371/journal.pntd.0013835 (PMC12747435; doi:10.1371/journal.pntd.0013835)

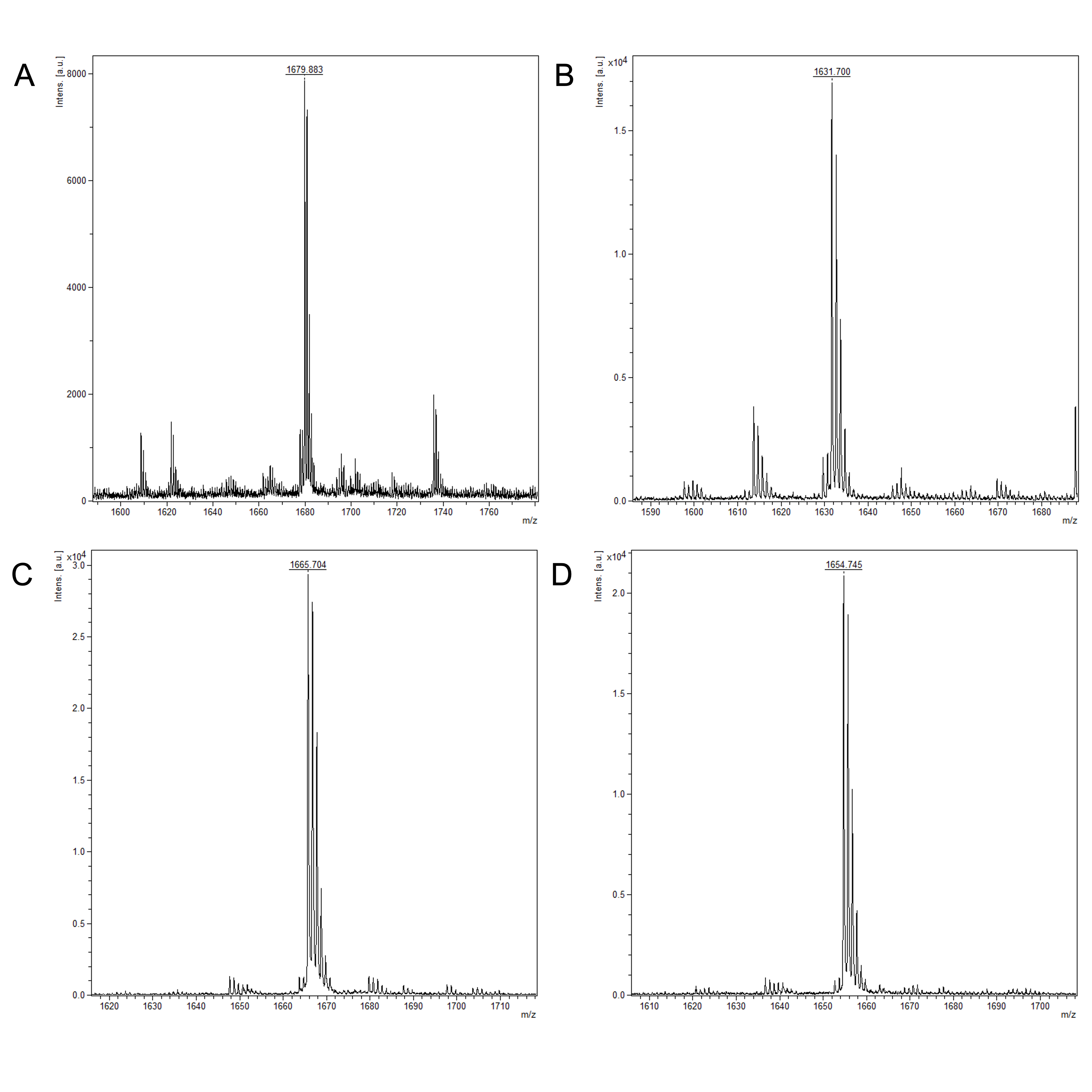

Supplement: S1 Fig — The data were obtained by MALDI-TOF/TOF for NK4 (A), NK6 (B), NK8 (C) and NK9 (D), synthesized in soluble form. (TIF) [file pntd.0013835.s001.tif]

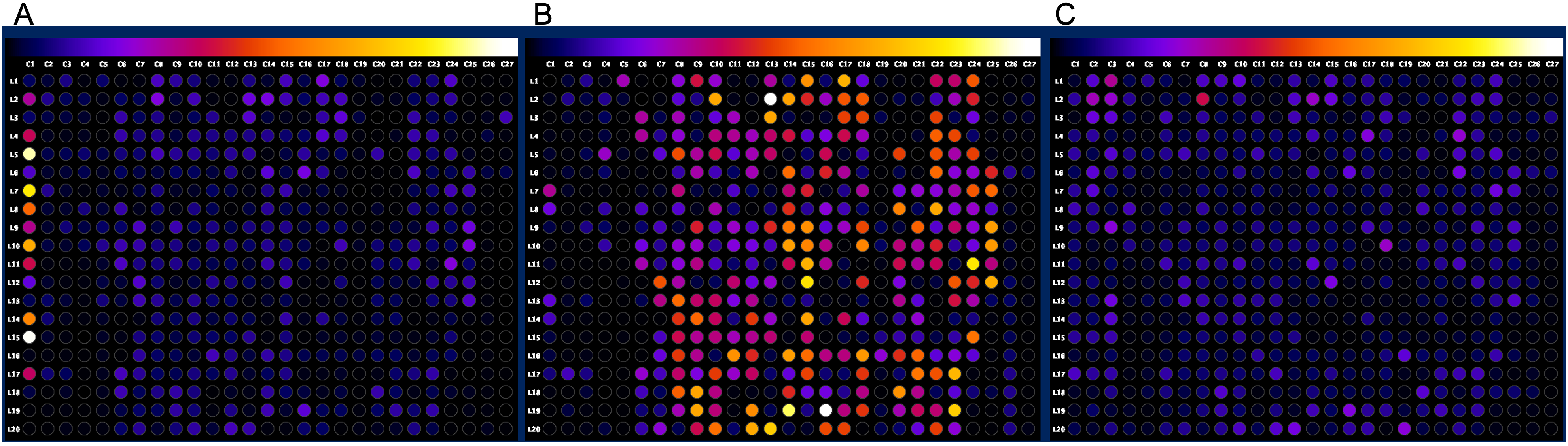

Supplement: S2 Fig — No peptides on this membrane met the selection criteria (values above the cut-off for T. cruzi-infected individuals and below for negative and VL samples). Panel A shows individuals in the chronic phase of T. cruzi infection, panel B shows uninfected donors, and panel C shows individuals with visceral leishmaniasis. Each dot corresponds to a peptide synthesized on the nitrocellulose membrane. Reactivity is displayed on a color scale ranging from black (low), orange (medium), to white (high). (TIF) [file pntd.0013835.s002.tif]

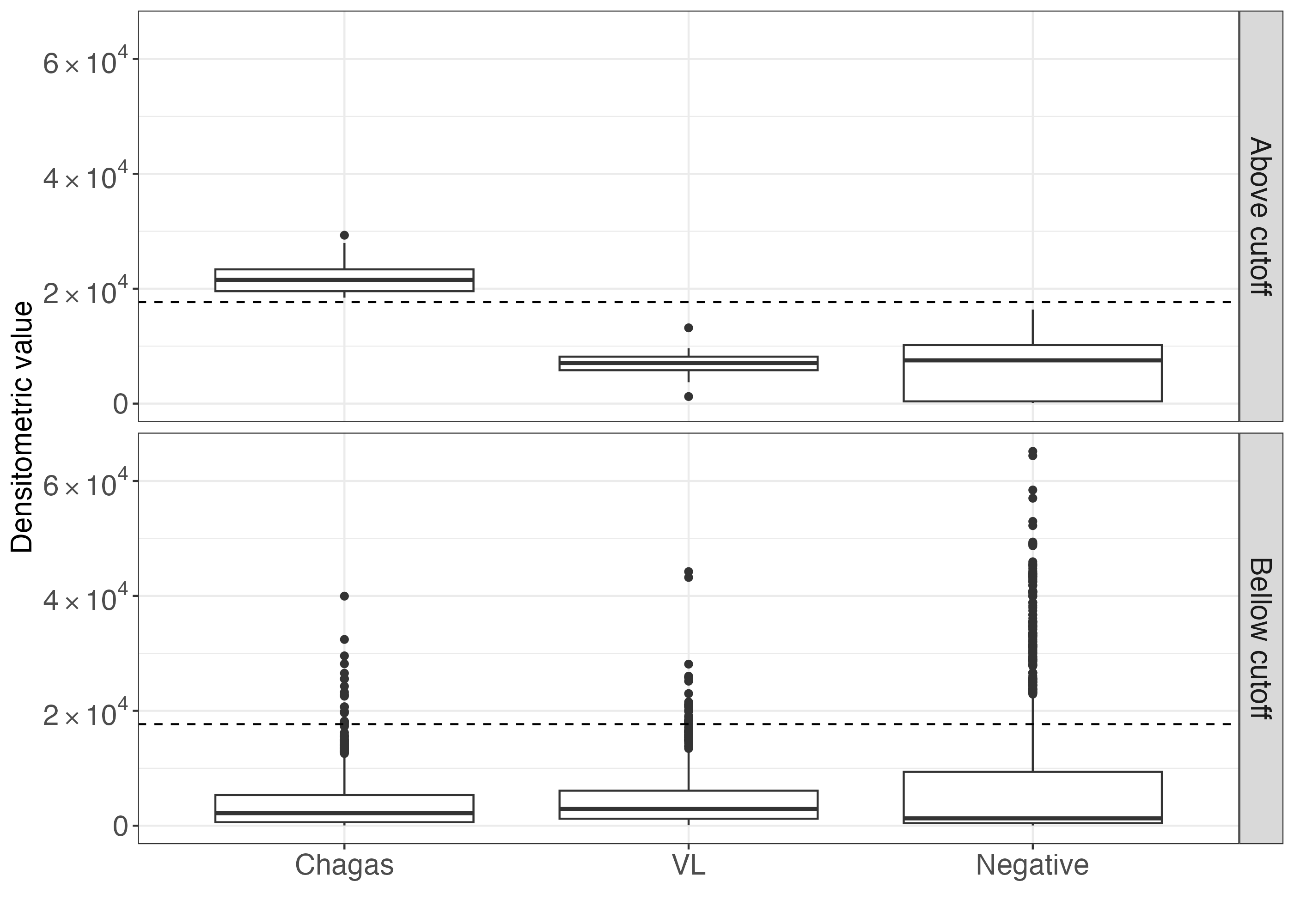

Supplement: S3 Fig — The top panel shows the peptides selected for further analyses, which display a consistent pattern of values above the cut-off in Chagas sera and below the cut-off in visceral leishmaniasis (VL) and negative sera. The bottom panel shows all remaining peptides tested. Each boxplot represents one sample group: individuals infected with T. cruzi (left), those with VL (center), and uninfected individuals (right). The dashed line indicates the cut-off value (17,672.72). Points appearing above or below the boxes represent outliers, including occasional negative or VL samples with values exceeding the cut-off. (TIF) [file pntd.0013835.s003.tif]

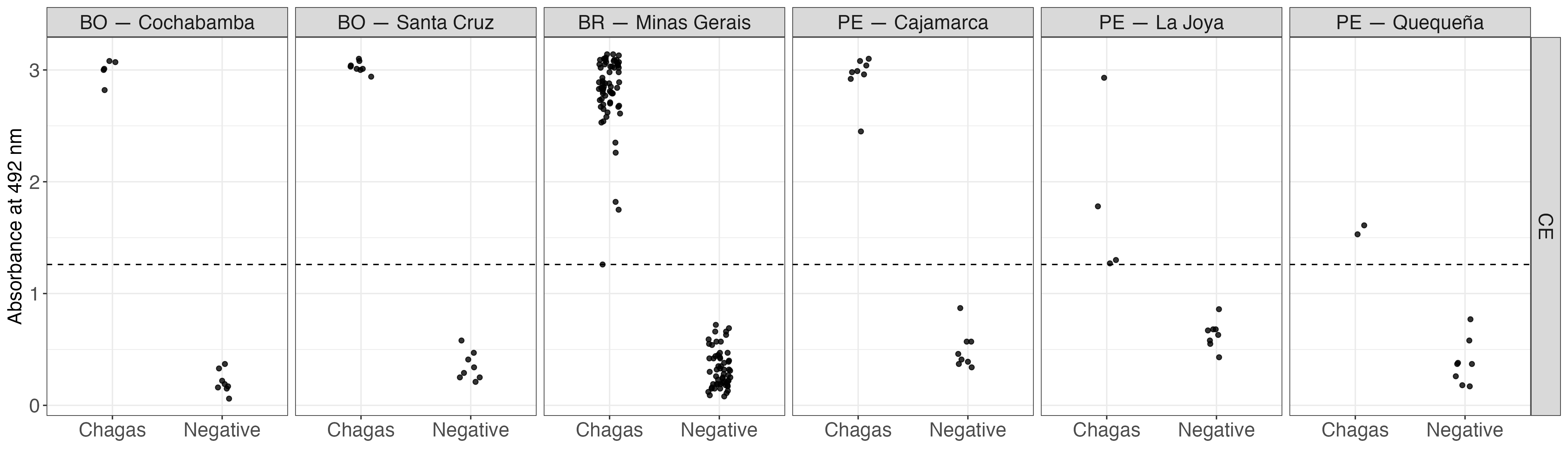

Supplement: S4 Fig — On the left side of each plot are individuals with CD (Chagas), and on the right side are uninfected individuals (Negative). The dashed line indicates the cut-off value based on the ROC curve and Youden’s index. (TIF) [file pntd.0013835.s004.tif]
